# Assessing Pathways to Carbon Neutrality in the Ceramic Sector: A Prospective Life Cycle Assessment under Energy System Projections and Technology Scenarios

**DOI:** 10.1021/acs.est.5c12177

**Published:** 2025-11-27

**Authors:** Ángel Galán-Martín, Richard Cabrera-Jimenez, Salvador Bueno-Rodríguez, Rosendo Jesús Galán-Arboledas, Gonzalo Guillén-Gosálbez

**Affiliations:** † Department of Chemical, Environmental and Materials Engineering, Institute of Biorefineries Research (I3B), 16747 University of Jaén, Campus Las Lagunillas s/n, 23071 Jaén, Spain; ‡ Institute for Chemical and Bioengineering, Department of Chemistry and Applied Biosciences, 27219 ETH Zürich, Vladimir-Prelog-Weg 1, 8093 Zürich, Switzerland; § Fundación Innovarcilla. Pol. Ind. El Cruce, C/Los Alamillos, 25, 23710 Bailén, Jaén, Spain

**Keywords:** ceramic industry, prospective life cycle assessment, decarbonization pathways, net-zero emissions, carbon capture and storage (CCS)

## Abstract

Ceramic manufacturing is a hard-to-abate industry given its intrinsic reliance on high-temperature operations and fossil-based thermal energy. Capitalizing on detailed industrial data, this study presents a prospective life cycle assessment of decarbonization pathways for ceramic brick production grounded in forward-looking energy system projections. Six technology adoption scenarios are evaluated for 2020 and 2050 under both current policy and net-zero trajectories. Results show that integrating heat pumps today for low-temperature drying can reduce the global warming potential (GWP) by up to 24% compared to continued natural gas use. Fuel-switching to hydrogen or biogas enables substantial reductions in the GWP of around 85% under net-zero conditions. Notably, coupling biogas combustion with carbon capture and storage (CCS) already achieves net-negative emissions, reaching −29.95 kg CO_2_-eq per tonne in 2020 and up to −73.05 kg by 2050, positioning it as a viable carbon dioxide removal strategy. However, several scenarios exhibit important trade-offs, including increased water consumption and land occupation, particularly in hydrogen and biobased pathways. Our findings underscore the need for integrated policies and multicriteria decision-making considering impacts beyond climate change to align energy transition and technology adoption, ensuring decarbonization strategies in the ceramic sector are both practical and sustainable.

## Graphical abstract

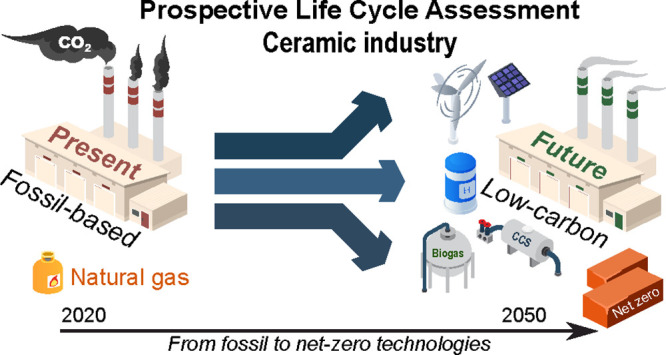

## 1 Introduction

The urgent need to mitigate climate change and achieve carbon neutrality by midcentury has driven increasingly ambitious global climate commitments.^1^ Achieving these targets requires a profound transformation across all sectors of the economy, particularly in industries heavily reliant on fossil fuels, which remain major contributors to global greenhouse gas (GHG) emissions.^2^ In 2022, the industrial sector alone accounted for approximately 38% of the global final energy consumption and 25% of the direct CO_2_ emissions, highlighting its central role in the decarbonization challenge.^3,4^ According to the Net Zero Emissions scenario of the International Energy Agency, process-related and combustion CO_2_ emissions in the industry must be reduced to near zero by 2050 to align with the Paris Agreement objectives.^5^

Within this context, energy-intensive industries emerge as strategic leverage points for accelerating climate action, given their high energy demands and dependence on carbon-intensive fuels—characteristics that often render them particularly hard to decarbonize.^6,7^ Alongside cement, steel, and glass, the ceramic sector belongs to this family of industries that rely on high-temperature heat, facing similar thermodynamic and technological constraints, which makes fuel substitution and electrification especially challenging.^5,8,9^ The ceramic industry, which plays a vital role in the construction and manufacturing sectors, exemplifies this challenge.^6^ It represents approximately 1% of the total industrial emissions under the EU Emissions Trading System (ETS), emitting approximately 20 million tonnes of CO_2_ annually in Europe.^10,11^ Its emissions are distributed across a highly fragmented sector composed mainly of small- and medium-sized enterprises (SMEs). As a result, ceramic installations represent nearly 10% of all industrial installations regulated under the ETS, posing considerable administrative and technological challenges for the large-scale implementation of decarbonization measures.^10^ In recognition of these structural limitations, the ceramic sector in countries such as Spain has benefited from special provisions under the ETS, including the free allocation of emission allowances and recognition as a sector at risk of carbon leakage. However, these preferential measures are being gradually phased out, increasing pressure on the industry to accelerate its decarbonization efforts.

The ceramic industry encompasses a wide range of products, from essential structural materials such as bricks, tiles, and roof elements to advanced ceramics with applications in the energy, electronics, and medical fields. Each product is manufactured through multistep processes that vary depending on the specific application, material requirements, and functional performance criteria.^12^ Despite product diversity, manufacturing lines follow a common process sequence involving raw material preparation (including grinding, mixing, and homogenization), shaping, drying, firing, and cooling and storage. Among these, drying and firing are the most thermally intensive stages. After the ceramic body is formed, drying is typically carried out at temperatures up to 100 °C. Firing is carried out in kilns operating typically at temperatures even above 1,000 °C, and while some waste heat is reused for drying, the process still depends on substantial thermal input, primarily from natural gas and coke. Consequently, the environmental performance of ceramic manufacturing is strongly dependent on the type and source of energy used throughout the process.^12,13^ Today, around 64% of the GHG emissions in the sector originate from fuel combustion, 17% from direct process emissions, and the remaining 19% from indirect and ancillary sources.^10^ These shares are broadly consistent with other hard-to-abate industries such as the steel, glass, and cement industries; however, ceramics differs in its high prevalence of small-scale plants and its combination of low- and high-temperature stages, which together create unique decarbonization pathways and constraints.^3,9^

The ongoing transformation of energy systems—driven by the increased integration of renewables and low-carbon energy carriers—offers a promising pathway toward decarbonizing ceramics. However, direct fuel substitution for high-temperature thermal applications remains economically and technically challenging. Despite these challenges, the ceramic industry is actively exploring and progressing toward reducing the environmental impact and GHG emission profile. Beyond future grid decarbonization and electrification, various technological approaches are being considered, each entailing distinct performance profiles and cost implications.^13^ Hence, their feasibility is often constrained—particularly for SMEs—by limitations in infrastructure and access to capital for large-scale retrofitting.^2,6,10,14^

In the short to medium term, viable decarbonization options include heat recovery, heat pumps (TRL 4–9) or microwave radiation for low-temperature drying processes, and fuel switching from coke to natural gas to hydrogen (TRL 5) in high-temperature kilns. Biomass and biofuels—derived from thermochemical or biochemical conversion of biomass—also represent potential low-carbon fuel alternatives when locally available. However, they typically have lower energy densities and combustion temperatures compared with fossil fuels. Direct combustion of biomass, syngas (TRL 2), or biogas or biomethane (TRL 5) can be used as substitutes for both drying and firing stages.^11^ Nonetheless, their adoption involves several technical, logistical, and economic challenges that must be addressed to enable their reliable and scalable integration into ceramic manufacturing systems. Finally, another line of action focuses on breakouts to prevent the release of CO_2_ generated through the integration of carbon capture and storage (CCS) technologies, which are currently at high TRLs (8–9) but are particularly difficult to implement due to the small to medium size of the enterprises.^15^ CCS may provide medium- to long-term solutions to mitigate and compensate process-related emissions, potentially enabling net-zero or even net-negative emissions in the sector when capturing and storing biogenic CO_2_.^7^

Despite growing interest in low-carbon ceramic manufacturing, there remains a notable gap in the literature regarding the comprehensive environmental assessment of decarbonization alternatives. Life cycle assessment (LCA) studies remain limited in the field. Most existing research focuses on tiles and sanitaryware—identifying hotspots such as firing and drying and recommending energy efficiency improvements and the use of recycled materials.^16–19^ Few studies have examined the climate-change-related impacts of traditional versus alternative bricks.^20^ Some recent studies have explored the integration of renewable energy^21^ and waste valorization,^22^ contributing to a broader understanding of circular economy opportunities in the sector. However, these studies are often conducted in isolation and lack a comprehensive perspective that captures the broader environmental implications—beyond climate change—of transformative technological pathways. Moreover, all of these studies narrowly assess current production systems without integration of future scenarios or system-level interactions. In contrast, recent analyses of other hard-to-abate industries (e.g., cement, steel, and chemicals) have highlighted both common technological opportunities and recurring challenges for high-temperature decarbonization.^9,23–26^ Yet, comparable prospective assessments for the ceramics sector remain scarce, particularly in a forward-looking context that reflects future developments in the energy system and technology development, leaving a gap in connecting ceramics to this wider body of knowledge contributing to strategic planning and policy support.

To address these knowledge gaps, we conduct here a novel prospective life cycle assessment (p-LCA) of decarbonization pathways in ceramic brick manufacturing. Unlike previous analyses, this work combines detailed operational data from real facilities with forward-looking energy system scenarios, which allows us to capture how technological transitions and energy decarbonization interact over time. This methodological framework, applied here for the first time to the ceramics sector, enables the analysis of multiple environmental dimensions beyond climate change while accounting for sector-specific constraints. In particular, we compare multiple scenarios involving retrofitting conventional processes with emerging low-carbon technologies benchmarked against a baseline natural gas-fired plant. Our scenario design reflects the unique structural and technological characteristics of the ceramic industry—its SME-dominated and decentralized structure, the coexistence of low- and high-temperature stages, and the strong link between product quality and firing conditions, all of which constrain or enable different decarbonization routes compared with other high-temperature sectors. By evaluating both present practices and prospective technological pathways, this study offers a comprehensive evaluation of environmental performance, extending beyond climate change to provide a deeper understanding of the trade-offs and cobenefits involved in transitioning toward a more sustainable ceramic industry. By situating ceramics within the broader family of hard-to-abate industries, this work also clarifies where sector-specific challenges and opportunities align with or diverge from those identified in other high-temperature sectors.

## 2 Methodology

This section outlines the methodological approach adopted to assess the environmental performance of decarbonization pathways in ceramic brick manufacturing. The study follows the principles and framework of LCA as defined by the ISO 14040 and 14044 standards,^27,28^ adopting a prospective LCA perspective (p-LCA) that integrates future energy system developments and emerging technological options.^29,30^

Figure 1 provides an overview of the analyzed brick production system under study and a schematic representation of the implementation of the p-LCA framework implemented. The foreground system is built on detailed, site-specific operational data collected from real ceramic brick manufacturers in Spain. The background system incorporates energy supply and material data sets from the Ecoinvent v3.9.1 database,^31^ modified to reflect future developments through the *premise* open-source toolbox.^29^ To conduct the prospective analysis, the background inventories were modified using the *premise* tool, based on projections from the REMIND integrated assessment model (IAM), a global multiregional model that couples energy system, macroeconomic, and climate dynamics to generate long-term scenarios for evaluating policy impacts and technology pathways.^32,33^ Specifically, two long-term scenarios were selected under the shared socioeconomic pathway 2 (SSP2 – “Middle of the Road”) narrative, each aligned with a different representative concentration pathway (RCP). The first, SSP2–None, reflects a continuation of current policies with moderate climate action, while the second, SSP2–RCP1.9, represents a stringent mitigation scenario consistent with achieving carbon neutrality by midcentury. This methodological framework enables a robust and forward-looking LCA that captures both present operational scenarios and future energy transitions. In the next sections, we detail the four key phases followed in conducting the LCA approach—goal and scope definition, life cycle inventory analysis, impact assessment, and interpretation—according to the standards.

**Figure 1:**
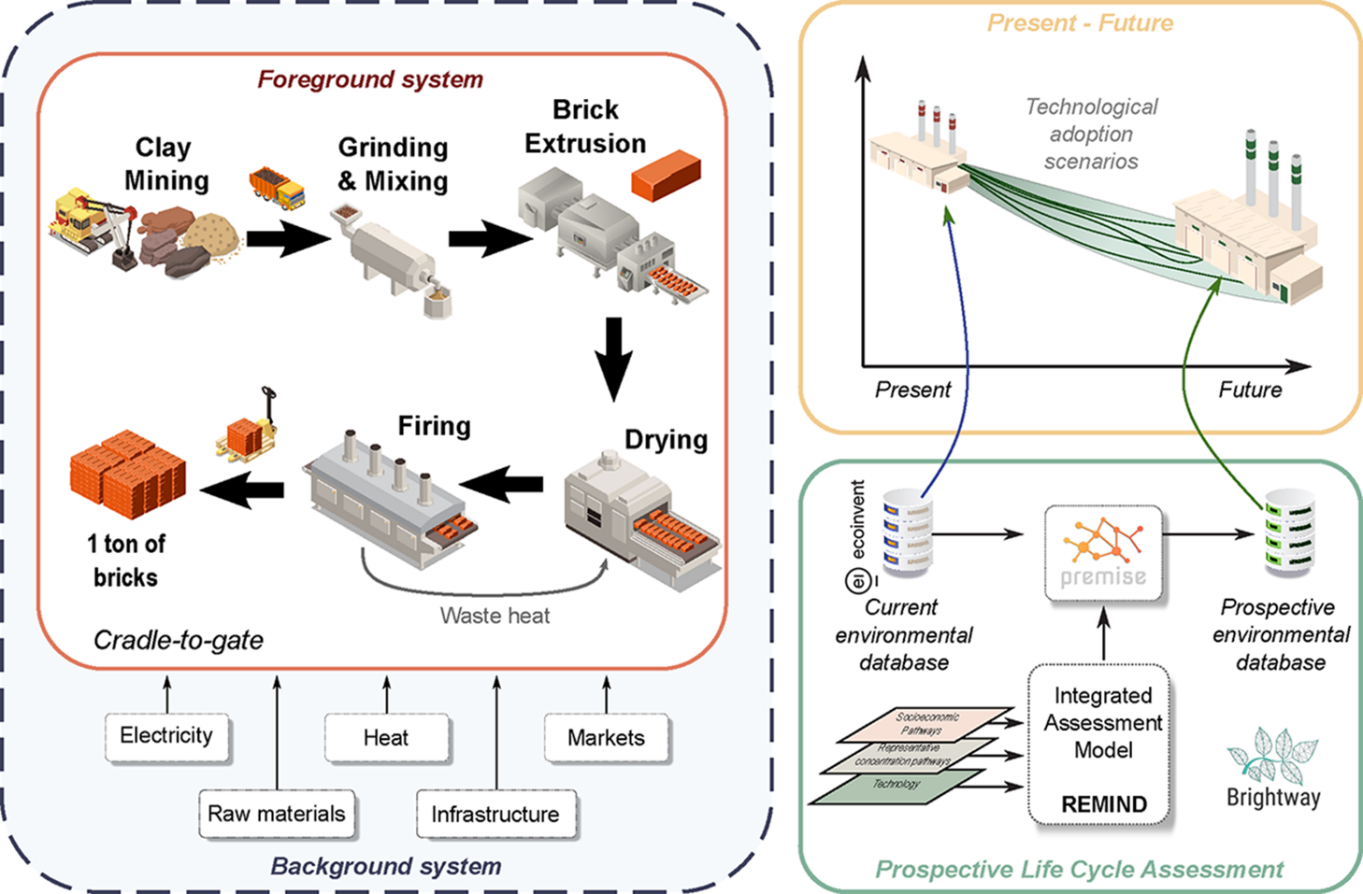
System boundaries of ceramic brick manufacturing and schematic representation of the p-LCA framework applied in this study.

### 2.1 Goal and Scope Definition

The primary goal of this study is to evaluate and compare the environmental performance of multiple decarbonization pathways in the context of ceramic brick manufacturing using a p-LCA approach. The methodology adheres to the principles and requirements outlined in ISO 14040 and ISO 14044 standards.^27,28^ This approach enables the analysis of future-oriented scenarios that reflect the integration of emerging low-carbon technologies and anticipated changes in energy systems.

The functional unit (FU) of the study is defined as one tonne of fired ceramic bricks delivered at the factory gate. This FU allows for consistently quantifying material and energy inputs, emissions, and environmental impacts across all modeled scenarios. Furthermore, this FU aligns with the requirements established in the environmental product declaration (EPD) framework for construction products, as defined by the EN 15804+A2 standard,^34^ which ensures relevance and comparability with other LCA studies and product declarations within the construction sector.

The system boundaries were defined following a cradle-to-gate approach, encompassing all processes from raw material extraction to the final brick product ready for transport (see Figure 1). This includes the quarrying and transporting of clay and supplementary materials to the production site, followed by raw material preparation activities, such as grinding, mixing, and homogenization. The shaped brick bodies are then formed through extrusion and cutting, after which they undergo drying through conventional convective methods or heat pump-assisted systems, depending on the specific scenario under evaluation (explained in section 2.1). Subsequently, bricks are subjected to high-temperature firing in kilns, typically operating between 900 and 1,100 °C—a stage representing the dominant source of energy consumption and emissions within the system. After firing, the bricks are cooled under controlled conditions and packaged for distribution. The system boundary excludes downstream stages, such as brick distribution, use, and end-of-life management. However, all upstream impacts, on-site energy use, and direct and indirect emissions are fully accounted. Notably, the study assesses a set of technological adoption scenarios that differ in the thermal and electrical energy sources supplied to the system, each representing a distinct decarbonization pathway. The specific characteristics of these scenarios are described in section 2.1.1.

#### 2.1.1 Technological Adoption Configurations

The design of technological scenarios was guided by the specific structural and operational characteristics of the ceramics industry, which differ from those of other hard-to-abate sectors. In ceramics, emissions are primarily associated with fuel combustion for thermal energy rather than process reactions, and the sector’s heterogeneous and SME-dominated structure limits the applicability of centralized, capital-intensive solutions. Additionally, the coexistence of low-temperature drying and high-temperature firing stages enables partial electrification options that are not feasible in sectors dominated by uniform, high-temperature processes. The selected technological adoptions, therefore, represent realistic, sector-specific technological pathways that capture both near- and long-term decarbonization opportunities, designed under a p-LCA framework that further integrates energy and policy developments projected through 2050.

Regarding the scenarios considered, our study comprises a reference configuration and a set of five alternative decarbonization pathways, each representing a distinct configuration of energy sources and technological interventions in ceramic brick manufacturing. The reference scenario reflects current ceramic brick production practices, while the alternative ones represent technologically feasible and promising decarbonization pathways.^6^ The scenarios are briefly described next.

- NG (Natural Gas - Reference Scenario): This is the reference scenario reflecting the current industrial practice, in which fossil natural gas is used exclusively to meet all thermal energy requirements in ceramic brick production. It is grounded in real operational data from existing industrial facilities located in Spain using clays with moderate carbonate contents (10–20% wt), thereby providing a representative baseline for comparison. This configuration serves as the reference baseline against which all alternative decarbonization pathways are compared across impact categories and timeframes.
- NG-HP (Heat Pump Scenario): This scenarios involves electrification of the drying stage by deploying high-efficiency heat pumps and replacing conventional gas-based drying systems. Heat pumps are assumed to operate with a coefficient of performance (COP) appropriate to industrial drying and are characterized by high technological maturity (TRL 4–9). Electrification through heat pumps entails upgrading electrical systems and air-handling units for low-temperature drying. This option reflects an early decarbonization opportunity unique to ceramics due to its distinct low-temperature drying stage.
- HYDROGEN (Hydrogen Scenario): In this scenario, natural gas is replaced with electrolytic hydrogen for both drying and high-temperature firing processes. Implementation requires installing hydrogen-compatible burners, on-site electrolysis systems, and appropriate storage and safety infrastructure. This scenario represents a midlevel maturity option (TRL 5), with significant decarbonization potential depending on the electricity source. Hydrogen is modeled as being produced via on-site water electrolysis powered by the Spanish electricity grid, reflecting current national energy conditions and prospective grid decarbonization trajectories.
- BIOGAS (Biogas Scenario): In this scenario, there is full substitution of fossil gas with biogas, covering both drying and firing stages. This scenario assumes the use of grid-injected biomethane, produced through decentralized facilities and upgraded via a combination of technologies (57% amine scrubbing, 16% pressure swing adsorption, and 26% membrane separation), achieving a heating value of 34.4 MJ/m^3^. Implementation may require adapting burners and fuel-supply systems and connecting to distribution networks. The scenario reflects a TRL of 5 and is consistent with the Spanish biomethane deployment strategy and national biogas roadmap, which prioritize the expansion of decentralized production capacity for industrial and energy applications. Biogas is assumed to be produced primarily from sewage sludge (59.80%), biowaste (36.40%), and a smaller fraction from livestock manure (1.70%), reflecting the most common feedstocks currently available in the national context. This decentralized supply approach reflects the compatibility of small-scale biogas production with the SME structure of the Spanish ceramics sector.
- NG+CCS (Natural gas with Carbon Capture and Storage): This scenario maintains natural gas as the thermal source but integrates postcombustion CO_2_ capture units at the kiln stage (assuming a 90% capture efficiency) and requires developing CO_2_ compression and transport infrastructure. Captured CO_2_ is transported 200 km and permanently stored in geological formations. It mitigates fossil CO_2_ and is thus considered a mitigation strategy. The technology is modeled at a TRL of 9, reflecting its expected availability for medium- to long-term industrial deployment. While CCS has high mitigation potential, its application in ceramics is constrained by plant scale and cost, which are explicitly considered in this scenario’s modeling assumptions.
- BIOGAS-CCS (Biogas with Carbon Capture and Storage Scenario): This scenario integrates postcombustion CO_2_ capture applied to the firing stage operated on biomethane (biogenic flue gases), also assuming 90% capture efficiency and geological storage. This scenario is categorized as a carbon dioxide removal (CDR) pathway, given the removal of biogenic carbon from the atmosphere. Like NG+CCS, it is modeled at a TRL of 9, acknowledging its emerging status and deployment challenges in the SME-dominated sector. This configuration explores the potential of ceramics to contribute to negative emissions through biogenic carbon capture—an opportunity rarely feasible in other high-temperature sectors.

All scenarios incorporate projections of future energy system developments, process efficiency, and the implementation of technologies by 2050, according to the integrated assessment modeling framework provided by REMIND and operationalized through the *premise* toolbox (Figure 1). These scenarios guide the modification of background data for each of the six modeled configurations—reference and five decarbonization pathways— facilitating the identification of trade-offs and cobenefits across multiple life cycle impact categories under a consistent, forward-looking framework.

### 2.2 Life Cycle Inventory and Prospective Modeling

The life cycle inventory (LCI) phase compiles all relevant input and output flows associated with the ceramic brick production system. The modeling distinguishes between foreground processes derived from primary data and background processes modeled based on established LCI databases. Foreground processes include all on-site operations directly related to ceramic brick manufacturing: raw material preparation, shaping, drying, firing, cooling, and auxiliary energy systems. Primary inventory data were collected from operational facilities located in Spain, reflecting actual production conditions. Due to nondisclosure agreements with participating parties, specific numerical values for the companies cannot be disclosed. The detailed life inventory tables can be found in Table S1 of the Supporting Information.

To build the inventories for the different scenarios, we relied on inventory data sets provided by the *premise* library for electrolytic hydrogen, biogas, and natural gas,^29^ while CCS scenarios were modeled based on Istrate and colleagues.^35^ To capture the influence of long-term transformations in energy systems, background inventories were adapted by using the *premise* tool, integrating projections from the REMIND model into the ecoinvent framework. Two scenarios from the REMIND model were considered under the SSP2 socioeconomic pathway: SSP2–None, representing the continuation of current policies and 3.5 °C of global temperature increase, and SSP2–1.9, a stringent mitigation pathway that limits global warming to 1.4 °C. These scenarios reflect different trajectories in electricity generation, fuel supply, and technology deployment within regional and national contexts. The modified background inventories were implemented in Brightway2,^36^ a Python-based LCA framework, enabling the integration of scenario-specific energy data with foreground industrial data in a transparent and reproducible way. This prospective modeling approach ensures that the environmental performance of decarbonization strategies is embedded within realistic future contexts.^30^

### 2.3 Life Cycle Impact Assessment

The life cycle impact assessment (LCIA) was conducted to quantify and compare the potential environmental impacts of each scenario across multiple configurations and timeframes. The entire LCI modeling and LCIA were performed using Brightway2 and Activity Browser v.2.9.0 to link foreground processes with background data. Environmental impacts were characterized at the midpoint level using the ReCiPe 2016 v1.03 method (hierarchist perspective).^37^ For focused discussion and interpretability, we selected five midpoint impact categories: global warming potential (GWP100, kg CO_2_-eq), fossil fuel potential (FFP, kg oil-eq), particulate matter formation potential (PMFP, kg PM_2_._5_-eq), water consumption potential (WCP, m^3^), and agricultural land occupation (LOP, m^2^ crop-eq). These indicators were chosen based on their policy relevance, connection to key environmental pressures, and specific implications for the ceramic manufacturing sector. GWP100 and FFP are core indicators in any decarbonization assessment, capturing the contribution to climate change and the depletion of fossil energy resources, respectively. PMFP and WCP address critical regional and operational dimensions. PMFP accounts for particulate matter emissions (PM_1__0_, PM_2.__5_) and precursors such as NO_*x*_ and SO_*x*_, which pose risks to human health and are subject to regulatory monitoring and reporting within the ceramic industry. WCP is particularly relevant in water-stressed regions and under transition pathways that may increase freshwater demand (e.g., those involving water-intensive electrolytic hydrogen production). Lastly, LOP is a key metric in biobased scenarios, reflecting the extent of land occupation associated with biomass supply chains and the potential impacts on biodiversity. All impact results were normalized per the FU of 1 tonne of fired ceramic bricks and are reported in both absolute terms and as relative differences from the reference scenario (NG). Where appropriate, results were disaggregated by life cycle stage (e.g., drying, firing, energy supply) to identify environmental hotspots and to support the interpretation of trade-offs and cobenefits across decarbonization pathways. The complete set of midpoint impact results across all ReCiPe 2016 categories is provided in Section S2 of the Supporting Information (Tables S3–S5).

## 3 Results and Discussion

### 3.1 Carbon Footprint Results

To evaluate the decarbonization potential of alternative technological pathways for ceramic brick production, we assessed the global warming potential (GWP) of each scenario across different timeframes and energy system configurations. Figure 2 presents the results for 2020 and projected values for 2050 under two future energy system assumptions: a Base scenario, reflecting the continuation of current policy trajectories (SSP2–None), and a Net Zero (NZ) scenario, aligned with stringent climate targets (SSP2–1.9).

**Figure 2:**
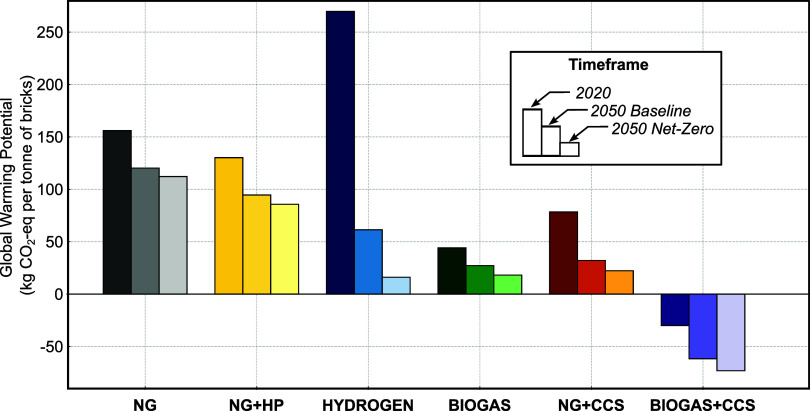
GWP (kg of CO_2_-eq per tonne of bricks) for selected technology adoption configurations in 2020 and projected for 2050 under two future scenarios: Base and Net Zero. Configurations include conventional fossil-based production using natural gas (NG), natural gas with electrified drying via heat pump (NG+HP), low-carbon fuels such as green hydrogen (HYDROGEN) and biogas (BIOGAS), and mitigation pathways involving carbon capture and storage (NG+CCS and BIOGAS+CCS).

The cross-scenario comparison reveals a general downward trend in GWP from 2020 to 2050, although the magnitude of the reduction varies substantially depending on the technology adopted. The NG scenario, representing current fossil-based practices that use natural gas to meet all thermal energy demands, shows the highest GWP in 2020 at 155.96 kg CO_2_-eq per tonne and remains relatively high even in 2050, dropping to 120.24 kg CO_2_-eq under the Base scenario and to 112.18 kg CO_2_-eq under NZ conditions. An exception is observed in the HYDROGEN scenario, which shows limited climate benefits in the short term by using the current electricity mix. Over time, the modest GWP reductions observed in the NG scenario illustrate that a continued reliance on carbon-intensive fuels, even with moderate grid decarbonization, is insufficient to meet climate goals. It also underscores the limited mitigation potential of maintaining the current infrastructure without switching to low-carbon energy sources or implementing additional abatement strategies. Moreover, persistent dependence on natural gas raises strategic energy concerns, particularly in the context of the EU’s objective to end reliance on imported fossil fuels and strengthen energy sovereignty. Similar challenges are evident in other energy-intensive sectors, such as cement and steel, where continued reliance on fossil-based fuels also undermines long-term decarbonization goals.^9^ However, unlike cement, ceramics lack significant process-related CO_2_ from calcination, meaning that fuel substitution and electrification play an even more central role in their mitigation trajectory.

In the short term, integrating heat pumps for low-temperature drying represents a technically viable and increasingly mature option. The NG+HP scenario, which combines natural gas for firing with electrified drying via high-efficiency heat pumps, reduces GWP from 130.19 kg of CO_2_-eq per tonne in 2020 to 94.55 kg of CO_2_-eq in 2050Base, and 85.67 kg of CO_2_-eq in 2050NZ—equivalent to reductions of 27 and 34%, respectively. While still dependent on fossil fuels, the partial substitution of thermal energy via electrification offers meaningful emission reductions, especially in regions where the electricity mix is rapidly decarbonizing. If implemented under current conditions, the shift from full natural gas use (NG scenario, 155.96 kg of CO_2_-eq per tonne) to the NG+HP configuration (130.19 kg of CO_2_-eq per tonne) would result in a 16.5% reduction in GWP, offering an immediate and actionable emission abatement measure while paving the way for further long-term technological upgrades. This benefit stems primarily from the reduced carbon intensity of drying heat, which drops from approximately 0.077 kg of CO_2_-eq/MJ when using natural gas to 0.017 kg of CO_2_-eq/MJ when powered by the Spanish electricity mix via high-efficiency heat pumps. As the grid continues to decarbonize, the environmental performance of such electrified systems is expected to improve further.

Replacing natural gas with hydrogen to meet high-temperature thermal demands is another pathway under consideration. In 2020, the HYDROGEN scenario shows the highest GWP overall at 269.72 kg CO_2_-eq per tonne, due to the use of grid electricity for hydrogen production, which in Spain is still largely fossil-based. This underscores the sensitivity of electrified fuels to the upstream electricity mix. However, the HYDROGEN scenario shows the steepest GWP reduction across the time horizon, falling to 61.37 kg of CO_2_-eq in 2050Base and just 16.12 kg of CO_2_-eq in 2050NZ—corresponding to 77 and 94% reductions, respectively. This substantial decline highlights hydrogen’s strong mitigation potential in deeply decarbonized energy systems and its infrastructure and supply chain challenges, including the need for renewable electricity, on-site generation or delivery logistics, and storage systems.^38^ These findings are consistent with cross-sectoral analyses that identify hydrogen as a critical but resource-intensive pathway for high-temperature industries,^39^ though ceramics may face comparatively fewer material compatibility constraints than steel or glass when adopting hydrogen combustion.^6,8^

On the other hand, the BIOGAS scenario—which involves substituting natural gas with biomethane for both drying and firing—demonstrates a moderate yet meaningful reduction in GWP in time, particularly under the net-zero (NZ) scenario. Specifically, emissions decrease from 44.10 kg of CO_2_-eq per tonne in 2020 to 18.20 kg of CO_2_-eq per tonne by 2050NZ (slightly higher than HYDROGEN), representing a 59% reduction. This improvement reflects the inherently lower carbon intensity of biogenic fuels and their reduced dependence on fossil-based energy inputs over the life cycle. However, the scenario remains constrained by upstream emissions from biomass cultivation, processing, and distribution, which prevent it from achieving net-zero results. Nevertheless, the BIOGAS scenario offers a compelling transitional mitigation option, enabling near-term GHG reductions while remaining compatible with future technological upgrades—such as the integration of CCS (scenario BIOGAS+CCS).^40^ In addition to its climate benefits, biogas deployment aligns with broader geopolitical and energy security goals. As outlined in the REPowerEU Plan^41^ and the recent EU roadmap to end dependence on Russian fossil fuel imports,^42^ scaling domestic biogas and biomethane production is a strategic priority.

Over the longer term, integrating CCS technologies and infrastructure could be pivotal in achieving deeper decarbonization in industries such as ceramic manufacturing.^38^ In the NG+CCS scenario, GWP falls from 78.38 kg of CO_2_-eq per tonne in 2020 to 32.09 kg of CO_2_-eq in 2050Base and 22.29 kg of CO_2_-eq in 2050NZ—an improvement of up to 72%, although effectiveness remains constrained by the carbon intensity of the upstream fuel supply. In contrast, the BIOGAS+CCS scenario achieves the lowest emissions intensity under the net-zero pathway, reaching −73.05 kg of CO_2_-eq per tonne of bricks by 2050. This net-negative performance is driven by the capture and permanent geological storage of biogenic CO_2_ released during biogas combustion, which more than offsets all associated upstream emissions. This results in net-negative emissions, positioning the scenario as a CDR pathway capable of offsetting emissions elsewhere. Moreover, the BIOGAS+CCS is not only a viable decarbonization strategy for hard-to-abate industrial sectors but also a candidate for generating carbon removal credits under emerging policy instruments, such as the EU Carbon Removal Certification Framework (CRCF).^43^ By meeting the criteria for durability, additionality, and monitoring, this pathway could potentially contribute to compliance or voluntary carbon markets, offering economic incentives for the early deployment and scaling of negative emission technologies in the ceramic industry.

### 3.2 Breakdown of Carbon Footprint

A detailed contribution analysis was conducted across all scenarios and timeframes to gain deeper insight into the underlying drivers of the GWP outcomes presented in the previous section (Figure 2). Figure 3 illustrates the breakdown of GWP by main life cycle components or input categories, such as fuels, electricity, and raw materials, while Figure 4 presents the corresponding breakdown by production stages, including drying, firing, and upstream processes. This dual-level disaggregation allows for a better interpretation of both technological and operational contributions to total GWP, thereby supporting the identification of key mitigation levers within each decarbonization pathway.

**Figure 3:**
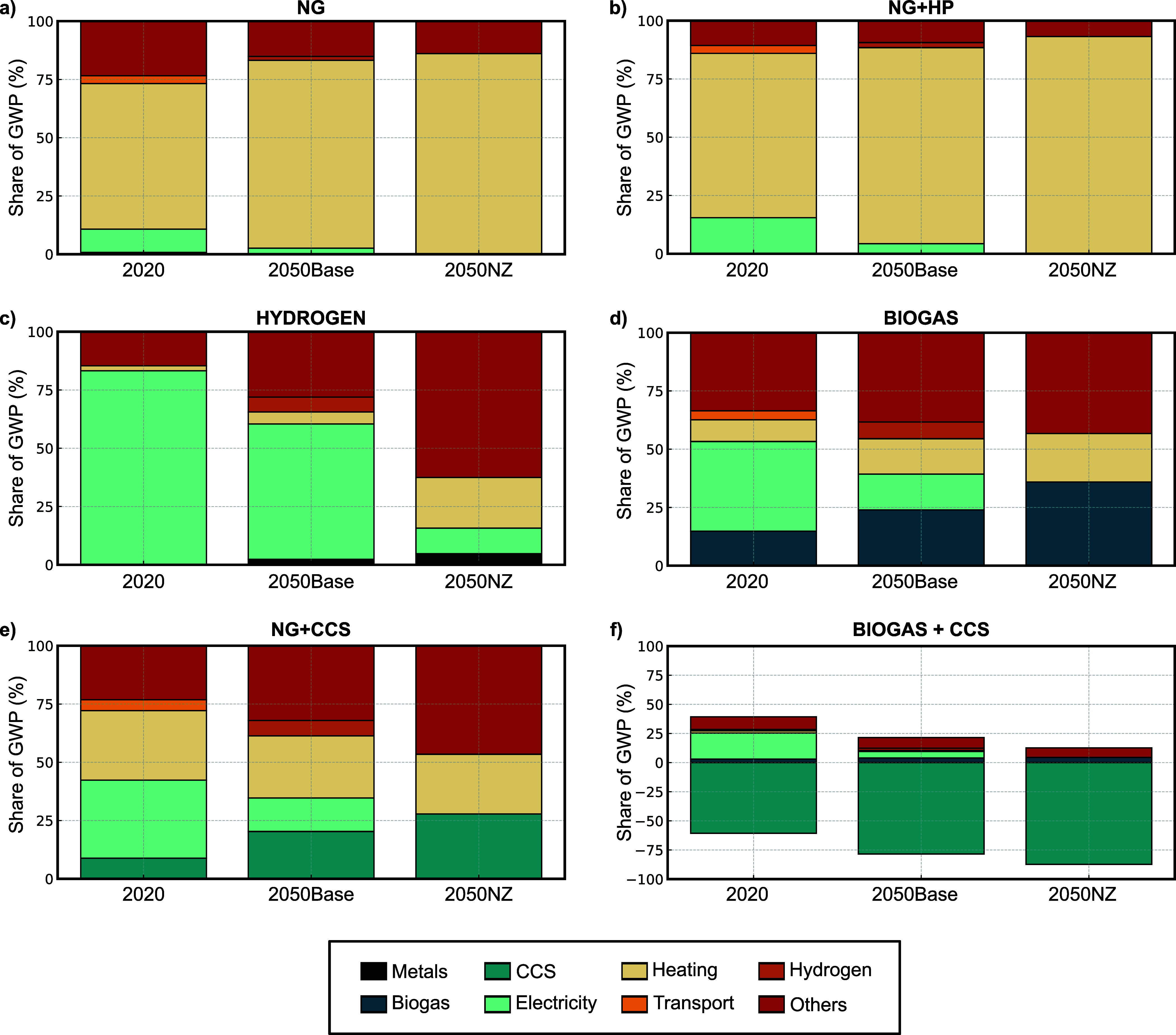
Normalized contribution of life cycle components to the GWP for six technological scenarios in ceramic brick manufacturing, evaluated for three timeframes (2020, 2050Base, and 2050NZ). Contributions are grouped into eight aggregated categories after applying a 1.5% cutoff to focus on the most relevant drivers. All contributions are normalized to 100%, except for the Biogas+CCS scenario, where the *y*-axis spans from −100 to +100% to accommodate net-negative emissions resulting from CCS.

**Figure 4:**
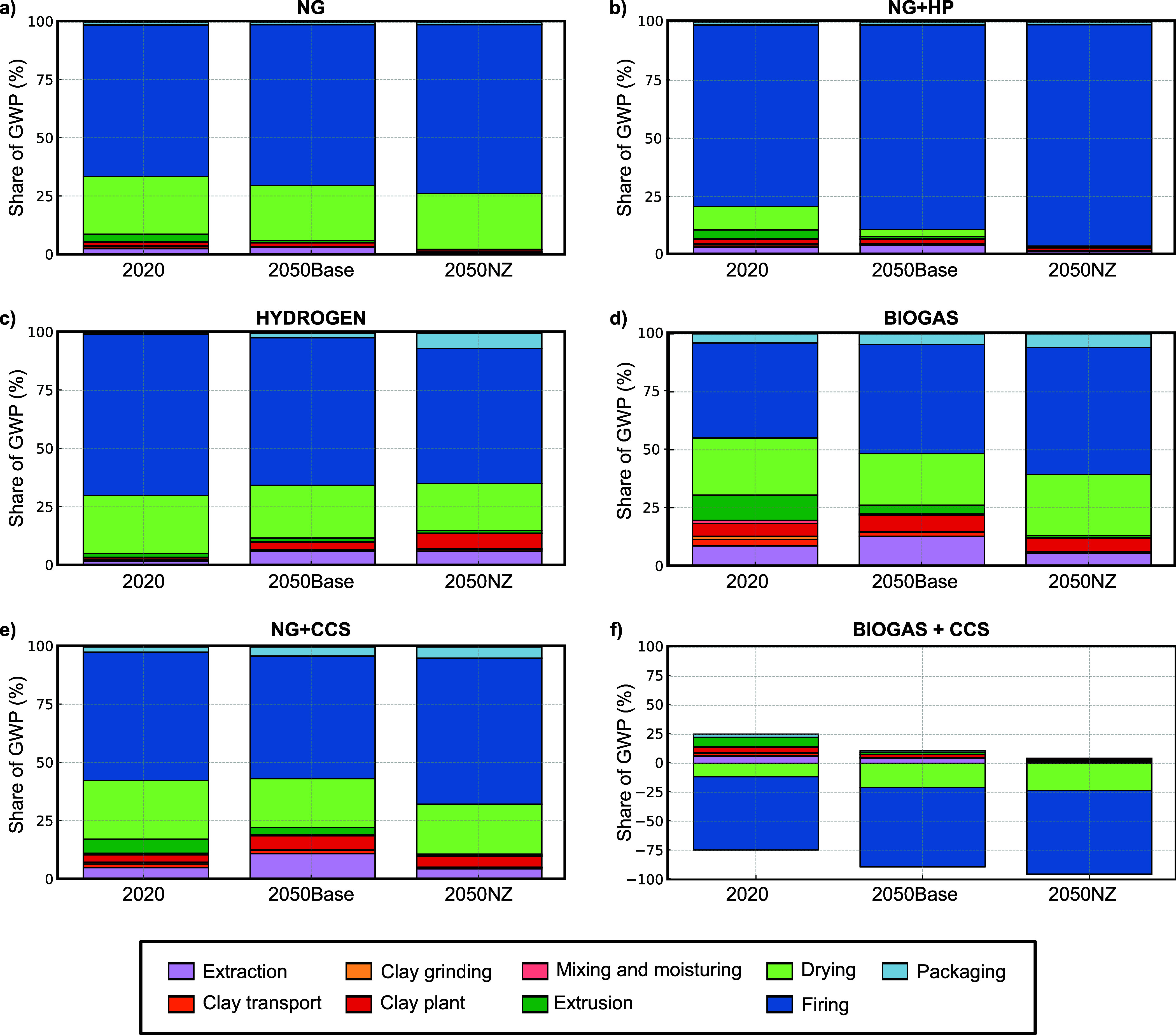
Normalized contribution of production activities (expressed as a percentage of total global warming) across six technological scenarios and three timeframes (2020, 2050Base, and 2050NZ). Contributions are grouped into nine categories, reflecting key stages of ceramic brick manufacturing. Values are expressed as percentages of the total GWP per scenario. All contributions are normalized to 100%, except for the Biogas+CCS scenario, where the *y*-axis spans −100 to +100% to reflect net-negative emissions enabled by CCS.

As shown in Figure 3, the contribution analysis by main life cycle components reveals clear differences in the relative importance of key life cycle elements grouped into eight categories: Metals, Biogas, CCS, Electricity, Heating, Transport, Hydrogen, and Others. These categories were derived by aggregating elementary flows and foreground process contributions, applying a 1.5% cutoff threshold to focus the analysis on the most influential contributors.

In fossil-based configurations such as NG and NG+HP (Figure 3, subplots a and b), the *Heating* category dominates GWP contributions, reflecting the central role of fossil natural gas for the thermal energy supply in current ceramic production processes. The contribution of *Heating* ranges between 78 and 83% across the three timeframes for the NG scenarios and from 70 to 85% in the NG+HP scenarios. This dominant share underscores the importance of targeting thermal energy provision, particularly in the firing and drying stages, as a priority in emission reduction strategies. Despite the partial electrification of the drying stage in the NG+HP scenario, natural gas remains the primary fuel for high-temperature firing, thereby sustaining *Heating* as the leading emission driver. Comparable dominance of thermal energy inputs is observed in cement kilns and primary steelmaking, where high-temperature combustion remains the primary driver of GHG emissions.^6,9^ However, ceramics differ in having a distinct low-temperature drying stage that is more amenable to electrification than that in cement or steel.

In contrast, for the alternative HYDROGEN and BIOGAS scenarios, the contributions from *Electricity* and *Biogas* become more prominent, particularly under the 2020 and 2050Base scenarios due to their upstream energy and processing burdens. Notably, the HYDROGEN scenario under 2020 exhibits a large share of emissions attributable to *Electricity* (approximately 83%), underscoring the high carbon intensity of grid-supplied power during the early transition period to produce electrolytic hydrogen. This dependency is critical as it reflects the sensitivity of hydrogen-based pathways to upstream electricity profiles. For the ceramic industry, the future deployment of hydrogen hinges on two main options: purchasing hydrogen from an external distribution network (a long-term strategy) or producing it on-site via electrolysis. In the latter case, renewable energy sources—such as solar photovoltaics and wind power—would offer the most climate-effective pathway. However, these options present practical challenges. On-site production from renewables projects requires substantial land or rooftop surface area, often limited or unavailable at existing ceramic facilities or in their immediate vicinity. As a result, a direct reliance on renewable electricity may not be feasible in many industrial contexts. In this regard, using electricity from the grid, while it progressively decarbonizes, represents a transitional solution to facilitate hydrogen adoption in the short- to medium term. Over time, the GWP in the HYDROGEN scenario decreases significantly, with a reduction of 77% from 2020 to the 2050Base scenario and 94% under the 2050NZ scenario (Figure 2). This trend aligns with decarbonization projections for hydrogen in cement,^23^ although the relatively smaller scale and distributed nature of ceramic plants may present both opportunities (easier piloting) and barriers (limited economies of scale) compared to larger industries. The progressive decarbonization of the electricity grid drives this reduction in the HYDROGEN scenario. For instance, the carbon intensity of electricity supply in Spain is projected to decline from approximately 0.26 kg of CO_2_-eq/kWh in 2020 to 0.059 kg of CO_2_-eq/kWh under the 2050Base and to 0.012 kg of CO_2_-eq/kWh under the 2050NZ scenario. As a result, the relative contribution of *Electricity* to the total GWP is reduced substantially, and other categories, such as *Metals*, *Heating*, and *Others,* emerge as residual contributors to the climate impact of the hydrogen-based production route.

Figure 3, subplot e, shows that in the NG+CCS scenario, where postcombustion CO_2_ capture and storage is applied to emissions from the firing stage, CCS contributes substantially to GWP reduction (especially under future scenarios). Nevertheless, *Heating* remains the largest positive contributor across all years, highlighting the need to decarbonize the thermal energy input to fully realize the mitigation potential of CCS. Notably, in 2020 and 2050Base, *Heating* accounts for more than 50% of the total impact, even as CCS effectively offsets a substantial portion of emissions. As shown in Figure 2, this NG+CCS scenario achieves considerable reductions in GWP relative to the conventional NG pathway, particularly under the 2050NZ scenario, where total emissions decline from 78.4 kg of CO_2_-eq per tonne in 2020 to 22.3 kg of CO_2_-eq per tonne in 2050, representing a reduction of approximately 71.6%. This highlights that the climate effectiveness of CCS depends on parallel decarbonization of the upstream energy supply, as its high energy demand and residual emissions (e.g., methane leakage and capture-related energy use) can still significantly impact the overall life cycle footprint. While these results are broadly consistent with CCS applications in cement and steel,^6,23^ ceramic faces unique challenges due to the prevalence of SMEs, which complicates investment in large-scale capture infrastructure.

Finally, the BIOGAS+CCS scenario presents a distinctive profile (Figure 3, subplot f). Here, the negative contribution of CCS (plotted below zero) can offset the positive emissions from other life cycle stages, leading to net-negative GHG emissions. This effect arises from the biogenic origin of the feedstock: CO_2_ initially captured by plants through photosynthesis is subsequently released during biogas combustion and then permanently sequestered via postcombustion capture. As such, the system effectively removes atmospheric CO_2_, reinforcing the role of biogenic carbon capture in a CDR strategy. Notably, the magnitude of the negative CCS contribution increases in the 2050Base and 2050NZ scenarios, reflecting the declining carbon intensity of the electricity/heating used to power the CCS process. As upstream emissions associated with energy inputs diminish, the relative impact of CCS becomes more favorable, further enhancing the net-negative performance of the Biogas+CCS configuration.

Next, we analyze the breakdown by specific life cycle activities within each technological scenario for ceramic brick production (Figure 4). *Firing* consistently emerges as the dominant contributor to GWP across all configurations, particularly under the baseline (2020) and intermediate decarbonization conditions (2050Base). This is primarily attributed to the thermal intensity of the firing stage, which relies heavily on direct fuel combustion and constitutes the most energy-intensive operation in the process chain. While major reductions are achieved through decarbonizing the *Firing* stage, the relative importance of noncombustion stages grows under low-emission scenarios, reinforcing the need for comprehensive mitigation strategies that address the whole process spectrum.

In the NG and NG+HP scenarios (relying on natural gas combustion), firing accounts for 101.9 kg of CO_2_-eq per tonne in 2020, representing approximately 65% of the total emissions in the NG scenario and 78% in the NG+HP scenario. *Drying* and *Extrusion* also contribute significantly to GWP, albeit to a lesser extent. *Drying* is the second most significant contributor in these configurations, with impacts reaching up to 38.9 kg of CO_2_-eq per tonne in the NG scenario for 2020 and 13.1 kg of CO_2_-eq per tonne in the NG+HP scenario, reflecting the considerable energy demand associated with moisture removal. Notably, the integration of heat pumps (HPs) in the NG+HP scenario reduces the GWP associated with the *drying* process by approximately 66% compared with the conventional NG configuration. These contributions reflect the cumulative impact of upstream electricity consumption and process heat demand. However, under future configurations with greater decarbonization efforts (e.g., 2050NZ), the relative contributions of these stages are substantially reduced due to the deployment of low-carbon energy carriers and enhanced energy efficiency throughout the process.

Under the HYDROGEN and BIOGAS scenarios (Figure 4, subplots c and d), the absolute contribution of *firing* and *drying* decreases due to the substitution of fossil fuels with cleaner energy carriers. However, in the BIOGAS scenario, its relative contribution to the total increases. In the BIOGAS case, *firing* contributes only 18.09 kg of CO_2_-eq per tonne in 2020, representing approximately 41% of the total GWP in that year, and 9.93 kg of CO_2_-eq per tonne under the 2050NZ condition, corresponding to ≈55% of the total emissions. In the HYDROGEN scenario, this value drops even further to 9.40 kg of CO_2_-eq per tonne in 2050NZ, accounting for approximately ≈58% of the total GWP. Even though upstream and auxiliary processes—such as *extraction*, *clay preparation*, and *packaging*— generally stay below 5.00 kg of CO_2_-eq per tonne each, they become more prominent in relative terms, collectively representing a notable contribution. This shift underscores the growing importance of background processes in low-emission configurations and highlights the need for comprehensive mitigation strategies that address both direct and indirect emissions along the entire ceramic brick production chain.

A notable shift is observed in the NG+CCS scenario (Figure 4, subplot e), where absolute emissions from *firing* decrease significantly due to the application of postcombustion carbon capture—from 43.47 kg CO_2_-eq per tonne in 2020, representing approximately 55% of the total GWP, to 14.05 kg CO_2_-eq per tonne in 2050NZ, corresponding to 63% of the total emissions. However, emissions from other upstream activities remain unchanged or are only slightly reduced, thereby increasing their relative share in the overall life cycle profile. For example, *drying* still contributes 19.76 kg of CO_2_-eq per tonne in 2020 and 4.80 kg of CO_2_-eq per tonne in 2050NZ, accounting for over 21% of the total GWP in the latter. Likewise, the relative importance of processes such as *extraction*, *clay preparation*, and *packaging* becomes more evident, underscoring the fact that while CCS can significantly mitigate direct emissions from fuel combustion, its impact on indirect and upstream sources is limited.

Finally, the BIOGASS+CCS scenario is the only configuration achieving net-negative emissions from the *firing* and *drying* stages. In 2050NZ, *firing* exhibits a contribution of −57.76 kg of CO_2_-eq per tonne, while *drying* accounts for −18.73 kg of CO_2_-eq per tonne, resulting from the combination of carbon-neutral biogenic inputs and CCS. This outcome is directly linked to the carbon-negative intensity of the renewable heating supply, which achieves net emissions as low as −0.044 kg of CO_2_-eq per MJ of thermal input. Nevertheless, all of the remaining activities—*extraction*, *clay transport* and *clay grinding*, the industrial *Clay plant*, *extrusion,* and *packaging*—remain positive although reduced over time, from 15.15 kg of CO_2_-eq per tonne in 2020 to 3.45 kg of CO_2_-eq per tonne in 2050NZ. Overall, the net-negative effect of −73.05 kg of CO_2_-eq per tonne in total requires efficient carbon capture technologies and decarbonizing the entire supply chain, including materials sourcing, handling, and auxiliary operations.

### 3.3 Environmental Trade-Offs of Decarbonization Pathways in Ceramic Production

The results presented in Figure 5 highlight the importance of evaluating decarbonization strategies from a multidimensional environmental perspective. While GWP remains a central metric for climate policy alignment, the broader set of indicators—FFP, PMFP, WCP, and LOP—reveals both trade-offs and cobenefits that are critical for a more comprehensive sustainability assessment.

**Figure 5:**
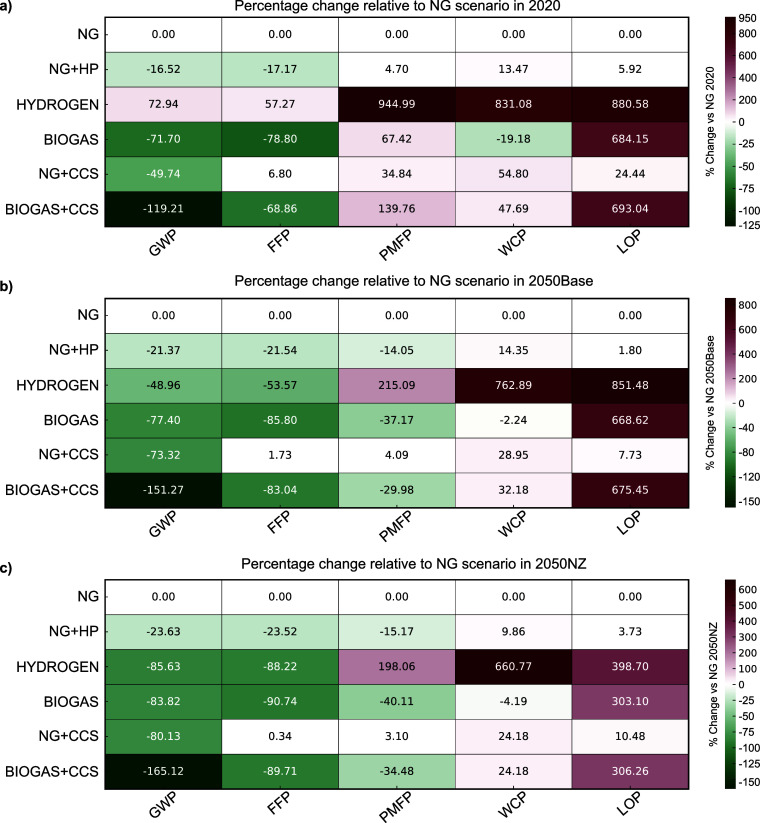
Heatmap showing the percentage change in selected environmental impact indicators relative to the natural gas (NG) reference scenario in the same year and for three timeframes: 2020 (top, subplot a), 2050 under the current policy baseline (middle, subplot b), and 2050 under Net Zero pathway (bottom, subplot c). Indicators include climate change (GWP100), fossil fuel depletion (FFP), particulate matter formation (PMFP), water consumption (WCP), and land occupation (LOP). Negative values (green) denote environmental improvements relative to NG, while positive values (red) indicate increased burdens.

The NG+HP scenario, which involves partial electrification through heat pumps, demonstrates consistent improvements in GWP (ranging from −16.52% in 2020 to −23.63% in 2050NZ) alongside moderate reductions in FFP and PMFP. Specifically, FFP declines steadily across all timeframes, from −17.17% in 2020 to −23.52% in 2050NZ. In contrast, PMFP initially increased in 2020 due to reliance on a fossil-intensive electricity grid, but it will improve by 2050 as fossil-based generation is phased out and replaced with cleaner alternatives. However, the scenario also exhibits slight increases in WCP and LOP. These unintended consequences may be linked to the electrification of thermal processes, which increasingly rely on a decarbonized grid with a large share of renewable energy technologies such as solar photovoltaics and biomass.

Integrating CCS into the current industry without natural gas fuel substitution (NG+CCS scenario) substantially reduces the GWP under long-term climate targets (Figure 2). However, it offers limited cobenefits in other categories, increasing impacts across all indicators and timeframes. Notably, in 2020, PMFP increases by 34.84%, while WCP and LOP rise by 54.80 and 24.44%, respectively, compared to the NG baseline. These effects can be primarily attributed to the additional energy demand for CO_2_ capture, compression, and handling, which can offset climate benefits and exacerbate other environmental pressures when sourced from a fossil-dominated grid. The dependency of CCS on low-impact energy inputs is thus a critical factor in determining its overall environmental performance. Trade-offs in PMFP are projected to be significantly alleviated in the future, declining from 34.84% in 2020 to only 3.10% in 2050NZ, largely due to the decarbonization of the electricity grid. Similarly, the burden-shifting observed in WCP and LOP is expected to diminish over time, though to a lesser extent. In 2050, NZ, WCP, and LOP will remain 24.18 and 10.48% higher than the NG reference.

In 2020, the HYDROGEN scenario exhibits the highest environmental burdens across all selected impact categories, including a 72.94% increase in GWP, and increases exceeding 800% in PMFP, WCP, and LOP compared with the NG baseline. These elevated impacts are primarily driven by the electricity-intensive nature of electrolytic hydrogen production, which when powered by a fossil-dominated grid results in substantial indirect emissions and particulate matter precursors. Under the 2050 Net Zero configurations, the HYDROGEN scenario substantially improves GWP and FFP (−85.63 and – 88.22%, respectively), reflecting the transition toward cleaner electricity sources. However, a notable burden-shifting effect persists, particularly toward WCP and LOP, which remain significantly higher than in the NG baseline —by 660.77 and 388.70%, respectively, under the 2050NZ scenario. This is primarily attributed to the substantial water demand for electrolytic hydrogen production (approximately 14 kg H_2_O per kg H_2_) as well as the large land footprint associated with renewable electricity generation, especially open ground solar photovoltaics and biomass (i.e., 34.5 and 3% respectively), used to power the electrolyzers.

Notably, the BIOGAS and BIOGAS + CCS pathways achieve the greatest GWP reductions across all timeframes (e.g., – 71.70% in 2020 for the BIOGAS scenario)—indicating the potential for net-negative emissions. However, these substantial climate benefits are accompanied by increases in WCP and, more critically, in LOP. For example, in 2020, the impact on LOP in the BIOGAS+CCS scenario is nearly seven times higher than that of the NG baseline. Although this impact decreases over time, it remains approximately three times higher in 2050NZ. These collateral effects are primarily driven by the agricultural processes required to cultivate and harvest biomass feedstocks, which are land-intensive and may also involve indirect environmental burdens such as habitat alteration and biodiversity loss. Specifically, the production and collection of sewage sludge, livestock manure, and particularly residual biomass involve land occupation for animal farming, nutrient management, logistics infrastructure, and water inputs related to biomass growth, cleaning, and anaerobic digestion operations. Although these feedstocks are often classified as wastes or residues, their valorization implies that environmental burdens are assigned to them. Importantly, including dedicated energy crops as biogas feedstock—though not assumed in this study—would magnify these trade-offs, given their direct requirements for arable land, irrigation, or fertilization application. As such, while biogas-based pathways deliver substantial climate benefits—particularly when coupled with CCS—their overall sustainability depends critically on governance mechanisms to secure waste- and residue-based feedstocks and to avoid indirect environmental burdens from energy crops. These results highlight a broader lesson: deep decarbonization in ceramics cannot be assessed solely based on climate indicators but must also account for potential trade-offs across multiple environmental dimensions.

In addition to environmental trade-offs, the feasibility of each decarbonization pathway is strongly influenced by techno-economic and infrastructural constraints associated with the SME-dominated industry. Electrification through high-efficiency heat pumps entails considerable upfront investment in equipment and electrical upgrades, yet offers long-term cost savings as electricity grids decarbonize and industrial-scale devices improve in efficiency and reliability.^8^ On the other hand, current energy prices, limited supply infrastructure, and uncertainties in future market development constrain fuel substitution with hydrogen or biogas/biomethane. In particular, the production and distribution costs of renewable hydrogen remain substantially higher than those of natural gas, while biogas availability is regionally limited by feedstock supply and competing uses.^39^ Finally, the integration of CCS, although effective for deep emission reductions, remains economically challenging for small and medium-sized ceramic facilities due to high capture costs and the absence of shared CO_2_ transport and storage networks.^44^

Beyond these economic barriers, structural characteristics of the ceramics sector—such as its SME-dominated composition and dispersed production sites—further limit access to capital-intensive technologies and centralized infrastructure. However, the progressive elimination of free allowances under the EU ETS increases the urgency for the sector to adopt viable low-carbon technologies and maintain competitiveness. Consequently, the realization of these pathways will depend not only on technological readiness but also on the implementation of coherent policy and industrial frameworks that lower capital barriers, promote regional energy integration, and support cluster-based deployment of low-carbon technologies. Instruments such as the EU CRCF and the REPowerEU Plan can play a decisive role in accelerating these transitions by incentivizing renewable gas production, electrified heat systems, and negative-emission technologies.

Placed in the broader context of other hard-to-abate industries such as cement, steel, and glass, many of the decarbonization strategies assessed here—electrification, hydrogen, biomass, and CCS—are shared across sectors. Yet ceramics exhibit distinctive features that shape both the feasibility and the sustainability of these options. Partial electrification of drying through high-efficiency heat pumps represents a near-term mitigation opportunity less available in sectors dominated by ultrahigh-temperature processes. The decentralized and SME-dominated structure of the ceramics industry may facilitate experimentation with decentralized biomass and electrification pathways while simultaneously creating barriers for the deployment of capital-intensive CCS. Furthermore, the strong sensitivity of product quality to firing conditions imposes operational constraints on fuel switching that differ from those observed in cement or steel manufacturing.

Ultimately, this study highlights that decarbonization in the ceramics sector cannot rely on a single technological lever. The sector-specific dynamics underscore that no single pathway provides a universal solution. Instead, a resilient transition will require a coordinated portfolio of complementary strategies and regionally adapted solutions supported by innovation, enabling policies, targeted financial instruments, and strategic infrastructure development. Future research should focus on demonstrating and scaling these technological options at pilot and industrial levels to validate their technical performance, economic feasibility, and system integration potential in real-world ceramic production environments. Hence, the analysis contributes not only to understanding the mitigation potential of the ceramic industry but also to clarifying where ceramics align with and where it diverges from other high-temperature industrial sectors. This dual perspective both strengthens the generalizability of decarbonization insights across hard-to-abate industries and highlights the unique challenges and opportunities of ceramics in the broader transition to net zero.

## Supplementary Material



## Data Availability

The data and code underlying this study are openly available in Zenodo at 10.5281/zenodo.17466490, which contains the life cycle inventory, scenario results, methodological framework, and main reference used for the prospective life cycle assessment of the ceramic sector.

## Notes

Detailed life cycle inventory data, along with additional environmental results that complement and expand upon the main findings (PDF)
