# Supplementary material for: Assessing Pathways to Carbon Neutrality in the Ceramic Sector: A Prospective Life Cycle Assessment under Energy System Projections and Technology Scenarios
Source: Environ Sci Technol. 2025 Nov 27;60(1):552–65. doi: 10.1021/acs.est.5c12177 (PMC12810240; doi:10.1021/acs.est.5c12177)
Supplement: Supplementary file 1 [file es5c12177_si_001.pdf]

Assessing Pathways to Carbon Neutrality in the Ceramic Sector: A Prospective Life Cycle Assessment Under Energy System Projections and Technology Scenarios

Ángel Galán-Martín[a*], Richard Cabrera-Jimenez[b], Salvador Bueno-Rodríguez[a], Rosendo Jesús Galán-Arboledas[c], Gonzalo Guillén-Gosálbez[b]

[a]Department of Chemical, Environmental and Materials Engineering, Institute of Biorefineries Research (I3B), University of Jaén, Campus Las Lagunillas s/n, 23071 Jaén, Spain

[b]Institute for Chemical and Bioengineering, Department of Chemistry and Applied Biosciences, ETH Zürich, Vladimir-Prelog-Weg 1, 8093, Zürich, Switzerland

[c]Fundación Innovarcilla. Pol. Ind. El Cruce, C/ Los Alamillos, 25, 23710 Bailén, Jaén, Spain

**Corresponding author: Ángel Galán-Martín*

Correspondence to: galan@ujaen.es

This document provides Supporting Information accompanying the content presented in the main article. It includes detailed life cycle inventory (LCI) data, along with additional environmental results that complement and expand upon the main findings.

Number of pages: 9

Number of tables: 5

S1 Life Cycle Inventory data

Table S1 presents the life cycle inventory (LCI) of the foreground system for ceramic brick production, developed based on detailed data obtained from real industrial operations. The inventory captures material and energy flows associated with each process stage, from raw material extraction to packaging. Whenever possible, specific machinery use, transport modes, and utilities consumption were quantified using primary data collected from collaborating facilities. Background data were sourced from the Ecoinvent database (version 3.9.1)[1] and from the literature, ensuring consistency with standard life cycle modeling practices. This inventory forms the basis for the environmental performance assessment of the baseline and alternative decarbonization scenarios.

Table S1. Inventory of elementary flows for the functional unit of 1 tonne of brick production, along with the corresponding datasets sourced from Ecoinvent v3.9.1[1].

Elementary flow	Amount	Activity name
Inputs:		
Extraction		
Clay, raw material (kg)	1,100.00	Clay, unspecified
Land occupation (m^2*year)	$18.37\ 10^{-2}$	Occupation, mineral extraction site
Land transformation (m^2)	$18.37\ 10^{-2}$	Transformation, to mineral extraction site
Loader (hour)	0.10	machine operation, diesel, < 18.64 kW, high load factor
Backhoe (hour)	0.18	machine operation, diesel, < 18.64 kW, high load factor
Truck (ton*km)	11.00	transport, freight, lorry 16-32 metric ton, EURO6
Tanker (ton*km)	0.11	transport, freight, lorry 3.5-7.5 metric ton, EURO6
Generator (hour)	$3.00\ 10^{-3}$	machine operation, diesel, >=18.64 kW and <74.57 kW, generators
Auxiliary group (hour)	$9.00\ 10^{-5}$	machine operation, diesel, >=18.64 kW and <74.57 kW, generators
Vehicle (km)	0.20	transport, passenger car, medium size, diesel, EURO5

Elementary flow	Amount	Activity name
Transport		
Truck from mine to plant (tkm)	6.60	transport, freight, lorry 16-32 metric ton, EURO6
Plant		
Infrastructure (unit)	$1.24\ 10^{-7}$	clay pit infrastructure
Clay grinding		
Electricity (kWh)	2.08	
Water (kg)	134.74	Tap water
Mixing and moisturizing		
Electricity (kWh)	2.00	*Alternative electricity sources depending on the scenario.
Water (kg)	57.74	Tap water
Extrusion		
Electricity (kWh)	18.16	*Alternative electricity sources depending on the scenario.
Drying		
Electricity (kWh)	22.04	*Alternative electricity sources depending on the scenario.
Heating (MJ)	467.62	#Alternative heat sources depending on the scenario.
Firing and cooking		
Electricity (kWh)	3.67	*Alternative sources depending on the scenario.
Heating (MJ)	1304.75	#Alternative heat sources depending on the scenario.

Elementary flow	Amount	Activity name
Packaging		
Pallet (unit)	$1.61\ 10^{-2}$	EUR-flat pallet
Plastic film (kg)	0.542	packaging film, low density polyethylene
Outputs:		
Bricks (ton)	1.00	-

*The source of electricity varies across scenarios and years, as defined using the premise tool, which integrates projections from the REMIND integrated assessment model[2] into the ecoinvent database. All scenarios account for anticipated changes in the energy system, process efficiency improvements, and technology deployment trajectories up to 2050. Specifically, two REMIND scenarios under the SSP2 socioeconomic pathway are used: (i) SSP2–None, reflecting a continuation of current policies and leading to approximately 3.5 °C of global warming (scenario 2050 Baseline), and (ii) SSP2–1.9, representing a stringent mitigation scenario consistent with a 1.4 °C warming target (scenario 2050 Net Zero). These projections influence the background electricity mixes and are aligned with the corresponding timeframes in the life cycle assessment.

#The source and composition of thermal energy used for heating processes vary across scenarios and timeframes. These differences reflect the adoption of distinct heat supply technologies—such as natural gas, biogas, hydrogen, and electricity—as defined in each decarbonization pathway. The evolution of heating-related background processes is modeled using the premise tool, which incorporates forward-looking projections from the REMIND integrated assessment model[2]. Specifically, the analysis considers two climate policy pathways: SSP2–None (current policy trajectory) and SSP2–1.9 (net-zero emissions trajectory). These assumptions influence the life cycle profiles of heat supply technologies across 2020, 2050 Base, and 2050 NZ timeframes. Detailed background LCI data for thermal energy carriers used in each scenario are provided in Table S2.

The background LCI data for thermal energy needed across the six decarbonization scenarios are compiled in Table S2. These scenarios include: (i) Natural Gas (NG), representing the current reference configuration; (ii) NG with Heat Pump (NG+HP), incorporating electricity-driven heat pumps for low-temperature drying; (iii) Hydrogen, involving complete fuel switching to electrolytic hydrogen for both drying and firing; (iv) Biogas, where fossil gas is fully substituted by grid-injected biomethane; (v) NG with Carbon Capture and Storage (NG+CCS), applying CO_2 capture to conventional fossil fuel use; and (vi) Biogas with CCS (Biogas+CCS), enabling net-negative emissions by capturing biogenic CO_2 during combustion. The LCI data were generated using the *premise* tool[3] to modify the ecoinvent v3.9.1 database, aligning background processes with forward-looking energy system projections derived from the REMIND integrated assessment model which are applied consistently to each scenario for the years 2020, 2050 Baseline, and 2050 Net Zero.

Table S2. Life cycle inventory data for the heating stage across the six technological scenarios considered in the study.

Scenario	Heating energy	Source
NG	Natural gas	[3]
NG+HP	Natural gas + electricity (Heat pump)	[3,6]
HYDROGEN	Hydrogen	[3]
BIOGAS	Biogas	[3]
NG+CCS	Natural gas with carbon, capture and storage	[3,4]
BIOGAS+CCS	Biogas with carbon, capture and storage	[3,4]

*The heat pump scenario assumes a coefficient of performance of 4.8, based on performance data from high-temperature industrial ammonia heat pumps (e.g., GEA Blu-Red Fusion series)

S2 Supplementary Results

This section provides detailed results for the full set of ReCiPe 2016 midpoint impact categories considered in the study[5]. Results are reported for each of the six technological scenarios (NG, NG+HP, Hydrogen, Biogas, NG+CCS, Biogas+CCS) and across the three temporal milestones (2020, 2050 Base, and 2050 Net Zero). These results offer a comprehensive view of the potential trade-offs and co-benefits associated with decarbonization pathways in the ceramic brick industry, highlighting variations not only in climate change impacts but also in categories such as ecotoxicity, resource depletion, particulate matter formation, and land and water use. The data support a multi-dimensional sustainability assessment and inform the interpretation of trade-offs beyond Global Warning Potential.

Table S3. Life cycle impact results for the eighteen ReCiPe 2016 midpoint indicators across the technology adoption scenario and for the timeframe 2020.

ReCiPe midpoint	Unit	NG	NG-HP	NG+CCS	Hydrogen	Biogas	Biogas+ CCS
Terrestrial acidification potential	*TAP (kg SO_2-eq)*	$1.44\ 10^{-1}$	$1.50\ 10^{-1}$	$2.09\ 10^{-1}$	$9.97\ 10^{-1}$	$1.43\ 10^{-1}$	$2.15\ 10^{-1}$
Global warming potential	*GWP (kg CO_2-eq)*	$1.56\ 10^{2}$	$1.30\ 10^{2}$	$7.84\ 10^{1}$	$2.70\ 10^{2}$	$4.41\ 10^{1}$	$-3.00\ 10^{1}$
Freshwater ecotoxicity potential	*FETP (kg 1,4-DCB-eq)*	2.11	1.96	2.65	$4.02\ 10^{1}$	2.01	2.85
Marine ecotoxicity potential	*METP (kg 1,4-DCB-eq)*	2.81E	2.62	3.51	$5.09\ 10^{1}$	2.66	3.74
Terrestrial ecotoxicity potential	*TETP (kg 1,4-DCB-eq)*	$2.11\ 10^{2}$	$2.02\ 10^{2}$	$2.33\ 10^{2}$	$1.96\ 10^{3}$	$2.14\ 10^{2}$	$2.42\ 10^{2}$
Fossil fuel potential	*FFP (kg oil-eq)*	$5.16\ 10^{1}$	$4.27\ 10^{1}$	$5.51\ 10^{1}$	$8.11\ 10^{1}$	$1.09\ 10^{1}$	$1.61\ 10^{1}$
Freshwater eutrophication potential	*FEP (kg P-eq)*	$1.02\ 10^{-2}$	$1.01\ 10^{-2}$	$2.15\ 10^{-2}$	$2.16\ 10^{-1}$	$9.01\ 10^{-3}$	$2.30\ 10^{-2}$
Marine eutrophication potential	*MEP (kg N-eq)*	$1.31\ 10^{-3}$	$1.42\ 10^{-3}$	$2.25\ 10^{-3}$	$1.65\ 10^{-2}$	$1.16\ 10^{-3}$	$2.32\ 10^{-3}$
Human toxicity carcinogenic	*HTPc (kg 1,4-DCB-eq)*	5.50	5.13	6.34	$2.10\ 10^{1}$	3.93	5.04
Human toxicity non carcinogenic	*HTPnc (kg 1,4-DCB-eq)*	$3.70\ 10^{1}$	$3.64\ 10^{1}$	$5.25\ 10^{1}$	$6.72\ 10^{-2}$	$3.64\ 10^{1}$	$5.72\ 10^{1}$
Ionising radiation potential	*IRP (kBq Co-60-eq)*	$1.26\ 10^{1}$	$1.77\ 10^{1}$	$2.01\ 10^{1}$	$1.60\ 10^{2}$	$1.17\ 10^{1}$	$1.99\ 10^{1}$
Land occupation potential	*LOP (m^2·year)*	1.57	1.67	1.96	4.06	2.83	3.63
Surplus ore potential	*SOP (kg Cu-eq).*	$1.30\ 10^{1}$	$1.29\ 10^{1}$	$1.31\ 10^{1}$	$1.54\ 10^{1}$	$1.23\ 10^{1}$	$1.25\ 10^{1}$
Ozone depletion potential	*ODP (kg CFC-11-eq)*	$2.94\ 10^{-5}$	$2.80\ 10^{-5}$	$3.54\ 10^{-5}$	$1.02\ 10^{-4}$	$3.58\ 10^{-5}$	$3.34\ 10^{-5}$
Particulate matter formation potential	*PMFP (kg $PM_{2.5}$-eq)*	$5.74\ 10^{-2}$	$6.01\ 10^{-2}$	$7.74\ 10^{-2}$	$3.20\ 10^{-1}$	$5.04\ 10^{-2}$	$8.40\ 10^{-2}$
Photochemical oxidant formation potential, human health	*HOFP (kg NMVOC-eq)*	$1.76\ 10^{-1}$	$1.65\ 10^{-1}$	$1.98\ 10^{-1}$	$6.00\ 10^{-1}$	$9.61\ 10^{-2}$	$1.38\ 10^{-1}$
Photochemical oxidant formation potential, ecosystems	*EOFP (kg NMVOC-eq)*	$1.94\ 10^{-1}$	$1.80\ 10^{-1}$	$2.17\ 10^{-1}$	$6.15\ 10^{-1}$	$1.01\ 10^{-1}$	$1.44\ 10^{-1}$
Water consumption potential	*WCP (m^3)*	$2.95\ 10^{-1}$	$3.35\ 10^{-1}$	$4.57\ 10^{-1}$	2.75	$2.39\ 10^{-1}$	$4.36\ 10^{-1}$

Table S4. Life cycle impact results for the eighteen ReCiPe 2016 midpoint indicators across the technology scenarios and for 2050 Baseline.

ReCiPe midpoint	Unit	NG	NG-HP	NG+CCS	Hydrogen	Biogas	Biogas+ CCS
Terrestrial acidification potential	*TAP (kg SO_2-eq)*	$6.46\ 10^{-2}$	$6.02\ 10^{-2}$	$9.21\ 10^{-2}$	$4.40\ 10^{-1}$	$9.08\ 10^{-2}$	$1.05\ 10^{-1}$
Global warming potential	*GWP (kg CO_2-eq)*	$1.20\ 10^{2}$	$9.45\ 10^{1}$	$3.21\ 10^{1}$	$6.14\ 10^{1}$	$2.72\ 10^{1}$	$-6.16\ 10^{1}$
Freshwater ecotoxicity potential	*FETP (kg 1,4-DCB-eq)*	2.19	2.16	2.61	3.45	2.25	2.89
Marine ecotoxicity potential	*METP (kg 1,4-DCB-eq)*	2.97	2.93	3.50	$4.33\ 10^{1}$	3.01	3.82
Terrestrial ecotoxicity potential	*TETP (kg 1,4-DCB-eq)*	$3.69\ 10^{2}$	$3.64\ 10^{2}$	$3.95\ 10^{2}$	$1.89\ 10^{3}$	$3.75\ 10^{2}$	$4.12\ 10^{2}$
Fossil fuel potential	*FFP (kg oil-eq)*	$4.35\ 10^{1}$	$3.41\ 10^{1}$	$4.42\ 10^{1}$	$2.02\ 10^{1}$	6.17	7.37
Freshwater eutrophication potential	*FEP (kg P-eq)*	$4.96\ 10^{-3}$	$5.14\ 10^{-3}$	$5.84\ 10^{-3}$	$4.10\ 10^{-2}$	$5.58\ 10^{-3}$	$6.33\ 10^{-3}$
Marine eutrophication potential	*MEP (kg N-eq)*	$6.43\ 10^{-4}$	$6.59\ 10^{-4}$	$8.15\ 10^{-4}$	$2.97\ 10^{-3}$	$6.79\ 10^{-4}$	$8.71\ 10^{-4}$
Human toxicity carcinogenic	*HTPc (kg 1,4-DCB-eq)*	5.06	4.84	5.45	$1.40\ 10^{1}$	4.15	4.70
Human toxicity non carcinogenic	*HTPnc (kg 1,4-DCB-eq)*	$3.10\ 10^{1}$	$3.05\ 10^{1}$	$3.53\ 10^{1}$	$4.45\ 10^{2}$	$3.33\ 10^{1}$	$3.96\ 10^{1}$
Ionising radiation potential	*IRP (kBq Co-60-eq)*	1.13	1.54	1.74	$1.39\ 10^{1}$	1.32	2.03
Land occupation potential	*LOP (m^2·year)*	1.59	1.62	1.72	4.47	2.98	3.40
Surplus ore potential	*SOP (kg Cu-eq).*	$1.25\ 10^{1}$	$1.25\ 10^{1}$	$1.26\ 10^{1}$	$1.52\ 10^{1}$	$1.23\ 10^{1}$	$1.24\ 10^{1}$
Ozone depletion potential	*ODP (kg CFC-11-eq)*	$1.38\ 10^{-5}$	$1.16\ 10^{-5}$	$1.46\ 10^{-5}$	$3.35\ 10^{-5}$	$2.84\ 10^{-5}$	$1.85\ 10^{-5}$
Particulate matter formation potential	*PMFP (kg $PM_{2.5}$-eq)*	$2.46\ 10^{-2}$	$2.30\ 10^{-2}$	$2.93\ 10^{-2}$	$1.40\ 10^{-1}$	$2.93\ 10^{-2}$	$3.19\ 10^{-2}$
Photochemical oxidant formation potential, human health	*HOFP (kg NMVOC-eq)*	$9.04\ 10^{-2}$	$7.77\ 10^{-2}$	$9.41\ 10^{-2}$	$2.85\ 10^{-1}$	$5.68\ 10^{-2}$	$6.33\ 10^{-2}$
Photochemical oxidant formation potential, ecosystems	*EOFP (kg NMVOC-eq)*	$9.64\ 10^{-2}$	$8.27\ 10^{-2}$	$1.00\ 10^{-1}$	$2.93\ 10^{-1}$	$5.97\ 10^{-2}$	$6.66\ 10^{-2}$
Water consumption potential	*WCP (m^3)*	$1.54\ 10^{-1}$	$1.76\ 10^{-1}$	$1.98\ 10^{-1}$	1.33	$1.50\ 10^{-1}$	$2.03\ 10^{-1}$

Table S5. Life cycle impact results for the eighteen ReCiPe 2016 midpoint indicators across the technology scenarios and for 2050 Net Zero.

ReCiPe midpoint	Unit	NG	NG-HP	NG+CCS	Hydrogen	Biogas	Biogas+ CCS
Terrestrial acidification potential	*TAP (kg SO_2-eq)*	6.11 10^{-2}	5.62E 10^{-2}	8.77 10^{-2}	4.17 10^{-1}	8.57 10^{-2}	9.83 10^{-2}
Global warming potential	*GWP (kg CO_2-eq)*	1.12 10^{2}	8.57 10^{1}	2.23 10^{1}	1.61 10^{1}	1.82 10^{1}	-7.31 10^{1}
Freshwater ecotoxicity potential	*FETP (kg 1,4-DCB-eq)*	2.54	2.55	3.03	3.63 10^{1}	2.59	3.31
Marine ecotoxicity potential	*METP (kg 1,4-DCB-eq)*	3.44	3.44	4.05	4.56 10^{-1}	3.48	4.37
Terrestrial ecotoxicity potential	*TETP (kg 1,4-DCB-eq)*	4.58 10^{2}	4.54 10^{2}	4.90 10^{2}	2.12 10^{2}	4.64 10^{2}	5.06 10^{2}
Fossil fuel potential	*FFP (kg oil-eq)*	4.13 10^{1}	3.16 10^{1}	4.14 10^{1}	4.86	3.82	4.25
Freshwater eutrophication potential	*FEP (kg P-eq)*	3.06 10^{-3}	2.98 10^{-3}	3.45 10^{-3}	2.84 10^{-2}	3.55 10^{-3}	3.69 10^{-3}
Marine eutrophication potential	*MEP (kg N-eq)*	5.90 10^{-4}	5.88 10^{-4}	7.39 10^{-4}	2.36 10^{-3}	6.10 10^{-4}	7.75 10^{-4}
Human toxicity carcinogenic	*HTPc (kg 1,4-DCB-eq)*	6.65	6.38	7.16	1.76 10^{1}	5.48	6.21
Human toxicity non carcinogenic	*HTPnc (kg 1,4-DCB-eq)*	3.34 10^{1}	3.30 10^{1}	3.80 10^{1}	4.52 10^{2}	3.54 10^{1}	4.18 10^{1}
Ionising radiation potential	*IRP (kBq Co-60-eq)*	9.71 10^{-1}	1.24	1.39	9.86	1.32	1.86
Land occupation potential	*LOP (m^2·year)*	3.03	3.14	3.34	1.04 10^{1}	4.31	4.94
Surplus ore potential	*SOP (kg Cu-eq).*	1.25 10^{1}	1.24 10^{1}	1.25 10^{1}	1.51 10^{1}	1.22 10^{1}	1.23 10^{1}
Ozone depletion potential	*ODP (kg CFC-11-eq)*	1.28 10^{-5}	1.04 10^{-5}	1.32 10^{-5}	2.40 10^{-5}	2.73 10^{-5}	1.69 10^{-5}
Particulate matter formation potential	*PMFP (kg $PM_{2.5}$-eq)*	2.36 10^{-2}	2.19 10^{-2}	2.80 10^{-2}	1.33 10^{-1}	2.77 10^{-2}	2.97 10^{-2}
Photochemical oxidant formation potential, human health	*HOFP (kg NMVOC-eq)*	8.70 10^{-2}	7.38 10^{-2}	8.97 10^{-2}	2.59 10^{-1}	5.21 10^{-2}	5.70 10^{-2}
Photochemical oxidant formation potential, ecosystems	*EOFP (kg NMVOC-eq)*	9.26 10^{-2}	7.84 10^{-2}	9.54 10^{-2}	2.66 10^{-1}	5.46 10^{-2}	5.97 10^{-2}
Water consumption potential	*WCP (m^3)*	1.44 10^{-1}	1.58 10^{-1}	1.78 10^{-1}	1.09	1.38 10^{-1}	1.79 10^{-1}

S3 References

(1) Wernet, G.; Bauer, C.; Steubing, B.; Reinhard, J.; Moreno-ruiz, E.; Weidema, B. The Ecoinvent Database Version 3 (Part I): Overview and Methodology. *Int. J. Life Cycle Assess.* **2016**, *3*, 1218–1230. https://doi.org/10.1007/s11367-016-1087-8.

(2) Baumstark, L.; Bauer, N.; Benke, F.; Bertram, C.; Bi, S.; Gong, C. C.; Dietrich, J. P.; Dirnaichner, A.; Giannousakis, A.; Hilaire, J. REMIND2. 1: Transformation and Innovation Dynamics of the Energy-Economic System within Climate and Sustainability Limits. *Geosci. Model Dev. Discuss.* **2021**, *2021*, 1–50.

(3) Sacchi, R.; Terlouw, T.; Siala, K.; Dirnaichner, A.; Bauer, C.; Cox, B.; Mutel, C.; Daioglou, V.; Luderer, G. PRospective EnvironMental Impact AsSEment (Premise): A Streamlined Approach to Producing Databases for Prospective Life Cycle Assessment Using Integrated Assessment Models. *Renew. Sustain. Energy Rev.* **2022**, *160*, 112311.

(4) Istrate, R.; Nabera, A.; Pérez-Ramírez, J.; Guillen-Gosalbez, G. One-Tenth of the EU's Sustainable Biomethane Coupled with Carbon Capture and Storage Can Enable Net-Zero Ammonia Production. *One Earth* **2024**, *7* (12), 2235–2249.

(5) Huijbregts, M. A. J.; Steinmann, Z. J. N.; Elshout, P. M. F.; Stam, G.; Verones, F.; Vieira, M.; Zijp, M.; Hollander, A.; van Zelm, R. ReCiPe2016: A Harmonised Life Cycle Impact Assessment Method at Midpoint and Endpoint Level. *Int. J. Life Cycle Assess.* **2017**, *22* (2), 138–147.

(6) GEA Refrigeration Germany GmbH. (2023). Product information, GEA Blu-Red Fusion. https://www.gea.com/en/products/chillers/gea-blu-red-fusion/
